# Acknowledgements to reviewers (2013–2014)

**DOI:** 10.1051/parasite/2015025

**Published:** 2015-10-02

The Editor depends heavily on reviewers in forming opinions about papers submitted to Parasite.

We are happy to formally thank the following scientists for their help, great effort and effectiveness in improving the quality of manuscripts.

Since January 2013, the rejection rate of Parasite has been about 50%; this list includes reviewers who evaluated papers which were not published.

For 2013 and 2014, our 292 reviewers from 51 countries are listed below. Names are in alphabetical order. Some have reviewed more than one manuscript.

 

Mohammd Abdigoudarzi (Iran)

Rashad Abdul-Ghani (Egypt)

Rob Adlard (Australia)

Peter H. Adler (United States)

Sargis Aghayan (Armenia)

Patrice Agnamey (France

Daniel Ajzenberg (France)

Fabio Akashi Hernandes (Brazil)

Okan Akhan (Turkey)

Sonia Almeria (Spain)

Bulent Alten (Turkey)

Alessandro Amarante (Brazil)

Karim Aoun (Tunisia)

Harry Archimede (France)

Herbert Auer (Austria)

Ali Ayadi (Tunisia)

Mehmet Fatih Aydin (Turkey)

Carlos Azevedo (Portugal)

Frédéric Baldacchino (France)

Thomas Barth (Germany)

Brigitte Bartholomot (France)

Eva Bártová (Czech Republic)

Jean-Claude Beaucournu (France)

Mustapha Benazzouz (Morocco)

Rosa Ma. del Refugio Bermudez Cruz (Mexico)

Frédéric Beugnet (France)

Ian Beveridge (Australia)

Sarah Bevins (United States)

Bhen Sikina Toguebaye (Senegal)

Pascal Boireau (France)

Marta Bombarova (Slovakia)

Sarah Bonnet (France)

Christian Bories (France)

Françoise Bouchet (France)

Franck Boué (France)

Nacim Bouheraoua (France)

Jérémy Bouyer (France)

Geoffrey Boxshall (United Kingdom)

Rodney A. Bray (United Kingdom)

Niel L. Bruce (Australia)

Magdaléna Brunanská (Slovakia)

Jacques Cabaret (France)

Simone Caccio (Italy)

Gioia Capelli (Italy)

Luis Cardoso (Portugal)

Graca Casal (Portugal)

Sibeli B. S. Cembranelli (Brazil)

Gabriela Certad (France)

Qijun Chen (China)

René Chermette (France)

Neil Chilton (Canada)

Bruno B. Chomel (United States)

Graham Clark (United Kingdom)

Helena Collgros (Spain)

Vince Connors (United States)

Miriam F. Cooperband (United States)

S. Z. Coskun (Turkey)

Mark Costello (New Zealand)

Thomas Cribb (Australia)

Liwang Cui (United States)

Bruno da Rocha Azevedo (United States)

John Dalton (Canada)

Marie-Laure Dardé (France)

Isaure de Buron (United States)

Johan F. De Jonckheere (Belgium)

Danielle Defaye (France)

Gunita Deksne (Latvia)

Pascal Delaunay (France)

Laurence Delhaes (France)

Jérôme Depaquit (France)

Peter Deplazes (Switzerland)

Anastasia Diakou (Greece)

Dimitar Dimitrov (Bulgaria)

Olgica Djurkovic (Serbia)

Philippe Dorchies (France)

Pierre Dorny (Belgium)

Pascal Drakulovski (France)

Gilles Dreyfuss (France)

Michael Dryden (United States)

Jitender P. Dubey (United States)

Michael Duchêne (Austria)

Jean Dupouy-Camet (France)

Lance Durden (United States)

Gérard Duvallet (France)

Chadli Dziri (Tunisia)

Samar N. El-Beshbishi (Egypt)

Agustin Estrada-Pena (Spain)

Loic Favennec (France)

Andy Fenton (United Kingdom)

Ignacio Ferre Pérez (Spain)

Raina Fichorova (United States)

John Fleming (United States)

Isabelle Florent (France)

William Font (United States)

Michel Franc (France)

Montserrat Gallego (Spain)

Martin Garcia Varela (Mexico)

Lamia Gargouri (Tunisia)

Claudia Gérard (France)

Mohamed Gharbi (Tunisia)

David I. Gibson (United Kingdom)

Cécile Ginane (France)

Patrick Giraudoux (France)

Jorge Gomez-Marin (Colombia)

Jorge F. Gonzalez (Spain)

David Gonzalez-Solis (Mexico)

Bruno Gottstein (Switzerland)

Sophie Goyard (France)

Katarzyna Goździk (Poland)

Tilmann Graeter (Germany)

Philippe Grellier (France)

Fréderic Grenouillet (France)

Norman Grim (United States)

Pascale Gueirard (France)

Patrick Guérin (Switzerland)

Neelima Gupta (India)

Carlos Gutierrez (Spain)

Hossein Hamidinejat (Iran)

Patrick Hamilton (United Kingdom)

Ralf Harbach (United Kingdom)

Shawgi Hassan (Sudan)

Michael Hastriter (United States)

Hilda Hernandez (Cuba)

Tony Holder (United Kingdom)

René Houin (France)

Sandrine Houzé (France)

Shaomin Hu (United States)

Yiau-Min Huang (United States)

Michael Huffman (United States)

Tine Huyse (Belgium)

Furhan Iqbal (Pakistan)

Kunihiko Izawa (Japan)

Yves Jackson (Switzerland)

Kym C. Jacobson (United States)

Philippe Jacquiet (France)

Aaron R. Jex (Australia)

F. Agustin Jimenez (United States)

Arlene Jones (United Kingdom)

Frans Jongejan (Netherlands)

Quentin Jossart (Belgium)

Kerstin Junker (South Africa)

Jean-Lou Justine (France)

Sukhbir Kaur (India)

Peter Kern (Germany)

Jamal Khalife (France)

Jean-Pierre Kinet (United States)

Jenny Knapp (France)

Janet Koprivnikar (Canada)

Mustafa Kose (Turkey)

Sabrina Krief (France)

Árni Kristmundsson (Iceland)

Delane Kritsky (United States)

Roman Kuchta (Czech Republic)

Katrin Kuhls (Germany)

Sandrine Lacour (France)

Kevin Lafferty (United States)

Irène Landau (France)

Gordon Langsley (France)

Wonchoel Lee (Korea)

Nils Leine (Norway)

Robert Lester (Australia)

Robert E. Lewis (United States)

Jun Li (China)

Marshall Lightowlers (Australia)

L. H. Susan Lim (Malaysia)

Renyong Lin (China)

David Scott Lindsay (United States)

Tim Littlewood (United Kingdom)

Badre lmimouni (Morocco)

Sean A. Locke (Canada)

Philippe Loiseau (France)

Bertrand Losson (Belgium)

Helder Louvandini (Brazil)

Liang Lu (China)

Hugo D. Lujan (Argentina)

Joseph W. Lynch (Australia)

Sutherland Maciver (United Kingdom)

Charles Mackenzie (United States)

Hervé Maisonneuve (France)

Laurence Malandrin (France)

Vincent Mallet (France)

Nathalie Mandonnet (France)

Sven Mangelinckx (Belgium)

Georges Mantion (France)

Laurence Marchat (Mexico)

Coralie Martin (France)

Alvaro Martinez Moreno (Spain)

Vesna Matijatko (Croatia)

Sheena McGowan (United States)

Concepta McManus (Brazil)

Antonio Menezes da Silva (Portugal)

Mercedes Mezo (Spain)

Terry Miller (Australia)

Laurence Millon (France)

Jordi Miquel (Spain)

Gholamreza Molavi (Iran)

Marcelo Molento (Brazil)

Scott Monks (Mexico)

Gisela R. A. Monteiro Marques (Brazil)

Jorge Morales-Montor (Brazil)

Serge Morand (France)

František Moravec (Czech Republic)

Sara Moutailler (France)

Yasen Mutafchiev (United States)

Lassad Neifar (Tunisia)

Cédric Neveu (France)

Anthony L. Newsome (United States)

Violaine Nicolas (France)

Karsten Noeckler (Germany)

Emiliano Ocampo (Argentina)

Lucia H. O’Dwyer (Brazil)

Annemarie Ohler (France)

Faith Osier (United Kingdom)

Domenico Otranto (Italy)

Harry Palm (Germany)

Carine Paraud (France)

Philippe Parola (France)

Maria Paulke-Korinek (Austria)

Adalberto Pérez de Leon (United States)

Anton Pérez-Rodriguez (Spain)

Bernard Pesson (France)

Romualda Petkevičiūtė (Lithuania)

Kurt Pfister (Germany)

Davies Mubika Pfukenyi (Zimbabwe)

Wojciech Piasecki (Poland)

Robert Poulin (New Zealand)

Edoardo Pozio (Italy)

Heather C. Proctor (Canada)

Eric Pulis (United States)

Karl Marx Quiazon (Philippines)

David Ranucci (Italy)

Christophe Ravel (France)

Richard Reithinger (United States)

Jean-Michel Repérant (France)

Vincent Robert (France)

Mike Rogan (United Kingdom)

Josée Roy (Canada)

Sadie F. Ryan (United States)

Guilermo Salgado-Maldonado (Mexico)

Rodrigo Sanabria (Argentina)

Julien Santi-Rocca (France)

Aurélia Saraiva (Portugal)

Brad Scandrett (Canada)

Gereon Schares (Germany)

Tomáš Scholz (Czech Republic)

Rolf K. Schuster (Germany)

Tengku Shahrul Anuar (Malaysia)

Jeffrey Shaw (Brazil)

Andrew Shinn (United Kingdom)

Didier Sicard (France)

Anne Silvestre (France)

Fernando Simon (Spain)

Bibhuti Singh (United States)

George Snounou (France)

Smaragda Sotiraki (Greece)

Isaia Sotiriadou (Germany)

Sabine Specht (Germany)

David Spratt (Australia)

Amir Steinman (Israel)

Balazs Szoor (United Kingdom)

Rachida Tahar (France)

Dennis Tappe (Germany)

David Taylor (United States)

Thomas H. Terrill (United States)

Richard Thacker (United Kingdom)

Jean-Marc Thellier (France)

Dave J. Thornton (United Kingdom)

Paul Torgerson (Switzerland)

Carine Truyens (Belgium)

Gerrit Uilenberg (France)

Gediminas Valkiunas (Lithuania)

Thirumalaisamy P. Velavan (Germany)

Veerle Versteirt (Belgium)

Isabelle Villena (France)

Christian Vivarès (France)

Petr Volf (Czech Republic)

Dominique A. Vuitton (France)

Martine Wallon (France)

Jianyang Wang (United States)

Andrew Williams (Denmark)

Thierry Wirth (France)

Anne-Sophie Woronoff (France)

Lihua Xiao (United States)

Cecilia Ximenez (Mexico)

Na Yang (China)

Hélène Yera (France)

Timothy Yoshino (United States)

Arnaldo Zaha (Brazil)

Eberhard Zeyhle (Kenya)

Yanlin Zhao (United States)

Xing-Quan Zhu (China)

Zhizhang Zhuang (China)

